# Tele-Yoga in Long Term Illness–Protocol for a Randomised Controlled Trial Including a Process Evaluation and Results from a Pilot Study

**DOI:** 10.3390/ijerph182111343

**Published:** 2021-10-28

**Authors:** Anna Strömberg, Ingela Thylén, Lotti Orwelius, Leonie Klompstra, Tiny Jaarsma

**Affiliations:** 1 Department of Health Medicine and Caring Sciences, Linköping University, 581 83 Linköping, Sweden; ingela.thylen@liu.se (I.T.); leonie.klompstra@liu.se (L.K.); tiny.jaarsma@liu.se (T.J.); 2 Department of Cardiology, Linköping University, 581 85 Linköping, Sweden; 3 Department of Anaesthesia and Intensive Care, Linköping University, 581 85 Linköping, Sweden; lotti.orvelius@regionostergotland.se; 4 Department of Biomedical and Clinical Sciences, Linköping University, 581 83 Linköping, Sweden

**Keywords:** heart failure, cardiac disease, mhealth, ehealth, tele-rehabilitation, yoga

## Abstract

Background: For people with long-term illness, debilitated by severe symptoms, it can be difficult to attend regular yoga classes. We have therefore developed a tele-health format of yoga that can be delivered in the home. The tele-yoga was co-designed with members of a patient-organisation, yoga-instructor, and IT-technician. It includes live-streamed group-yoga sessions twice a week and an app with instructions on how to self-perform yoga. Aim: To describe a study protocol for a randomised controlled trial (RCT) including a process evaluation and report on a pilot study evaluating method- and intervention-related components including feasibility, safety, and efficacy. Methods: Ten participants with heart failure aged between 41–76 years were randomised to tele-yoga (*n* = 5) or to the control group (*n* = 5). In the pilot study recruitment, enrolment, randomisation, and data collection of all outcomes including primary, secondary and process evaluation measures were tested according to the study protocol. Fidelity, adherence and acceptability to the tele-yoga group training and app use was determined. Safety was assessed by adverse events. Results: The pilot revealed that the methodological aspect of the protocol worked sufficiently in all aspects except for missing data in the physical test of two participants and one participant in the control-group that dropped out of the study at three months follow-up. The tele-yoga training did not lead to any adverse events or injuries, adherence of tele-yoga was sufficient according to preset limits. The tele-yoga intervention also showed some favourable trends of improvements in the composite-end point compared to the active control group. However, since data only was presented descriptively due to the small sample size, the impact of these trends should be interpreted carefully. Conclusion: Our pilot study showed promising results in feasibility, safety, and acceptability of the tele-yoga intervention. Some changes in the protocol have been made to decrease the risk of missing data in the measures of physical function and in the full-scale RCT now ongoing the results of the sample size calculation for 300 participants have included the estimated level of drop outs and missing data.

## 1. Introduction

Yoga is an ancient form of exercise, that in recent years has increased in popularity across all age groups. Yoga has a holistic approach that may target several physical and mental problems and symptoms from which people with long-term illness may suffer [1].

Several studies have demonstrated the positive effects of yoga on a number of health outcomes, such as improvements in symptoms of depression and anxiety [2], well-being, enhanced sleep quality [3] and cognition [4]. Physiological effects of yoga are decreased breathing and heart rate, decreased blood pressure, restored autonomic regulatory reflex mechanisms, and improved cardiometabolic health [1].

Medical yoga is a therapeutic form of Kundalini yoga with structured programs including postures, breathing exercises and meditation specifically targeting persons with long-term illness. Medical yoga has been shown to improve health-related quality of life and physical function as well as reduce anxiety and depression [1,5]. Medical yoga is mostly introduced and performed through face-to-face yoga classes delivered by health care professionals that are certified yoga instructors. However, for some people with highly symptomatic long-term illnesses e.g moderate-severe heart failure, it can be difficult to leave the home to attend regular yoga classes. Our group has previously shown that medical yoga can be an alternative or complement to established forms of exercise training [5]. While the medical yoga intervention was found to be feasible, and adherence was good, we observed that many prospective participants refrained from participating on account of it being too time-consuming and exhausting to leave their home twice a week to travel (sometimes a considerable distance) to the site of the yoga-class [5]. Tele-health, defined as the delivery of health-related services via digital communication technologies can be used to provide yoga classes at home. In a previous feasibility study, this type of tele-health interventions with yoga has been called tele-yoga [6]. In the same tele-yoga project a qualitative analysis of patients experiences revealed that the intervention was acceptable and that the home-based aspect for highly symptomatic patients with heart failure and chronic obstructive lung disease was highly appropriate. However, the technology did need refinement [7]. When delivering tele-yoga, face-to-face contact between participants and the yoga instructor is removed. This may influence the experience, safety and outcomes of the medical tele-yoga. However, the goal with tele-yoga is to provide medical yoga of equal quality and safety as face-to-face but with improved access for all patients, also highly symptomatic and homebound patients, at a lower cost. Therefore, further studies of the feasibility, safety and efficacy of tele-yoga are warranted. This paper describes the co-design development of a tele-yoga intervention for persons with long-term illness, the protocol for a randomised controlled trial (RCT) with a mixed method process evaluation, and results from a pilot study exploring feasibility, acceptability, safety, and efficacy of tele-yoga with the same protocol.

## 2. Methods

### 2.1. Development of the Tele-Yoga Intervention

The tele-yoga intervention was developed in collaboration with the patient organisation Riksförbundet HjärtLung (https://www.hjart-lung.se/, accessed 23 October 2021), a certified medical yoga-instructor and an IT technician over a period of 12 weeks during early 2018. The tele-yoga intervention included (1) video communication via Zoom and (2) app installed on a tablet with a sim card and 4G broadband technology. After the initial testing, participants from the patient organisation (aged 70–93 years) completed a survey on their experiences of tele-yoga which focused on both the technology and the yoga. Based on this feed-back modifications of the tele-yoga sessions and app were performed in collaboration with participants from the patient organisation, the yoga instructor, IT technician and the research team. 

### 2.2. Study Protocol

The protocol is based on the CONSORT statement (http://www.consort-statement.org/ accessed 23 October 2021), TIDieR checklist [8] and SPIRIT statement (www.spirit-statement.org accessed 23 October 2021) and the process evaluation of complex interventions [9].

**Figure 1:**
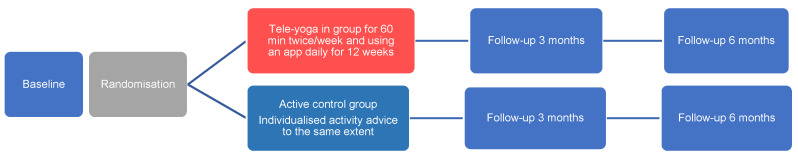
Flow chart of the study design with randomisation, intervention, and follow-up.

The study is a single-blind (evaluator blinded) parallel two-arm randomised controlled study with 1:1 distribution to intervention control group and a mixed method process evaluation. Data are collected at baseline measurement, and after 3 and 6 months, see Figure 1 The trial is registered in ClinicalTrials.gov, ID: NCT 03703609.

### 2.3. Study Participants

The study is recruiting participants from four different hospitals, one university hospital and three county hospitals in three different county councils/regions in the south-east of Sweden.

### 2.4. Inclusion Criteria

Being over the age of 18 years; diagnosed with a long-term illness and cared for at the department of cardiology or department of intensive care clinic for at least 48 h in the last 3–36 months; and clinically stable condition at the time of inclusion.

### 2.5. Exclusion Criteria

Inability to fill in questionnaires and/or to participate in the tele-yoga intervention due to severe physical or mental limitation based on chart review and screening interviews; expected survival of less than 6 months.

### 2.6. Hypothesis

The primary hypothesis is that medical tele-yoga significantly increases physical function and health-related quality of life and reduces symptoms of anxiety and depression in people with long-term illnesses compared to an active control group receiving individualised training advice to the same extent in terms of duration and frequency. The secondary hypothesis is that medical tele-yoga improves sleep, heart rate, blood pressure, and cognition in people with long-term illnesses significantly compared with an active control group receiving individual exercise advice to the same extent in terms of duration and frequency.

### 2.7. Research Questions Related to the Process Evaluation

#### 2.7.1. Health Technology Assessment

What effect has structured introduction and access to tele-yoga for people with long-term illnesses on healthcare consumption and QALYs compared with an active control group receiving individual training advice?

What is the participants’ willingness-to-pay for the intervention?

#### 2.7.2. Patient Experiences and Preferences

What are the experiences of tele-yoga in people with long-term illnesses?

#### 2.7.3. Exercise Motivation, Fidelity, Adherence and Social Selectivity

How are exercise motivation, fidelity and adherence for people with long-term illnesses affected when participants receive a structured introduction and access to tele-yoga for 12 weeks?

Is there a difference between participants who accept and decline study participation and those who drop out compared to those completing the study?

### 2.8. Intervention Group (Tele-Yoga)

#### 2.8.1. Group Yoga Sessions

Participants perform a 60-min tele-yoga session in a group of approximately 10 study participants twice a week, conducted by a livestreamed instructor via a videotelephony software program (Zoom Video Communications Inc., San Jose, CA, USA) on a tablet at home. Each participant performs a total of 20–24 yoga instructor-led group tele-yoga sessions over a 12-week period. Certified yoga instructors deliver a therapeutic form of Kundalini yoga (https://mediyoga.se, accessed 23 October 2021). The movements in this practice are slower than in traditional forms of yoga. The starting position is often sitting but can also be standing or lying on a yoga mat. Two standardised programs are used. Each of which includes a combination of breathing exercises, physical postures, meditation, and relaxation.

Programme-I (total of 58 min) where participants will learn and practice long-deep breathing. This programme includes two physical postures spinal flex and Sat Kriya. It includes the heart meditation and Kirtan kriya finger meditation.

Programme-II (a total of 53 min), where participants will practice deep breathing and ‘breath of fire’ exercises. Physical postures will focus on the spine with several exercises that gently turn the back in different directions. Meditation includes breathing meditation with inhalation through the left nostrils and exhalation through the right nostril, and pulse meditation where participants both concentrate on their breathing and feeling their pulse. Relaxation, warm-up, and cool-down will be delivered in both programs. The rationale for having two different programs is that they include different postures and meditations and provide variety in the sessions for participants. Previous experience from medical yoga studies [5,10] have shown that a two-week period for each program is optimal with enough time to master the postures and meditations. These two programs have been tested in a previous study of people with heart failure [5] and atrial fibrillation [10]. Before and at the end of each session, participants will have the opportunity to discuss their experiences or address questions to the instructor online. The instructor monitored participation in the yoga group to ensure that all participated in the predetermined number of sessions.

#### 2.8.2. Yoga App

The tablet also includes an app with instructions (text, pictures, and sound files) for yoga positions, breathing and meditation. Participants are encouraged to practice yoga at home individually with a goal of 50 min per week. For example, one session for at least 10 min a day 5 days/week.

### 2.9. Active Control Group

In addition to the usual treatment and information on rehabilitation and daily activities, participants in the control group receive an exercise program that corresponds to the intervention group in time and effort. The training program is given by a physiotherapist or a nurse and is based on individual functional ability, the patient's preferences, and current international guidelines for physical activity. Participants in the control group are encouraged to exercise daily to the same extent as the intervention group, i.e., physical activity equivalent to 60 min for 2 days a week and 10 min for the other 5 days. Examples of activities can be walking, biking, going to the gym. To compensate for the extra attention received by the intervention group from the instructor via tele-yoga group, the control group's patients will be telephoned or have SMS contact (the participant chooses the type of contact) with a physiotherapist or nurse after 2, 4, 8, and 12 weeks.

### 2.10. Data Collection

All data collection is performed in an out-patient clinic at the hospitals. At baseline before randomisation, and after 3 months, physical function (6 min walk test [11]), dynamic balance/mobility (Sit to stand test [12]), self-selected walking speed (Gait speed [13]), blood pressure, pulse , breathing and cognitive ability measured by Montreal Cognitive Assessment (MoCA) [14] are measured by blinded data collectors. Participants also fill in a battery of questionnaires including questions about socio-demographic variables, yoga experience and technology and user-friendliness, health-related quality of life measured by Euroqol 5-Dimensions (EQ-5D) and thermometer (https://euroqol.org/, accessed 23 October 2021) and two general health question (#35 and 36) from RAND-36 [15], symptoms of anxiety and depression with the Hospital anxiety and depression scale [16] (HADS), sleep with the minimal insomnia symptom scale (MISS) [17] and the Exercise Motivation Index [18] Completing the questionnaires electronically on a tablet takes approximately 30 min. Physical activity are objectively monitored for one week at baseline and after 3 and 6 months with an Activity Monitor (Actigraph - https://actigraphcorp.com/, accessed 23 October 2021). At the end of the study, healthcare utilisation are recorded from the medical charts. All participants in the tele-yoga group are interviewed at baseline, and at 3 and 6 months using a semi-structured interview guide.

The primary outcome variable is a composite end-point. The composite weighed end-point consists of physical ability (6 min walk test), health-related quality of life (EQ-5D) and symptoms of anxiety and depression (HADS).

### 2.11. Qualitative Analysis

Tape-recorded and transcribed semi-structured interviews on expectations of the tele-yoga sessions after randomisation at baseline, and experiences of the tele-yoga sessions after 3 and 6 months and analysed using inductive content analysis according to Elo and Kyngäs [19].

### 2.12. Health Technology Assessment

Costs of the intervention and health care utilisation as well as QALYs are calculated.

## 3. Pilot Study

The pilot study investigated the feasibility of the design of the RCT study with special focus on recruitment, randomisation, enrollment, fidelity and collection of data for all outcome variables including the process evaluation. A sample size of 10 participants were estimated to be sufficient. The study protocol as described in Section 2.2, Section 2.3, Section 2.4, Section 2.5, Section 2.6, Section 2.7, Section 2.8, Section 2.9, Section 2.10, Section 2.11 and Section 2.12 was complimented by the CONSORT extension for randomised pilot and feasibility trials [20]. Only data on safety, feasibility, fidelity and the primary and secondary outcomes were included in the analysis of the pilot study.

A chart review was conducted based on diagnoses lists from one cardiology and one intensive-care unit. Eligible patients were sent written information by post and later invited to participate through a telephone call. Participants gave written informed consent and were included in data collection at baseline and after 3 months from March to June 2018. In the protocol described for a planned full-scale RCT a 6 month follow-up will also be conducted.

### 3.1. Feasibility of Interventions (Tele-Yoga and Active Control Group)

Feasibility was measured as fidelity defined as if the study groups (both the tele-yoga and active control groups) received the intervention or instructions exactly as described in the study protocol. Adherence to group-teleyoga during the 12 weeks of intervention was measured by registration of participation in the Zoom meeting done by the yoga instructors. The active control group was called by a physiotherapist or nurse after 2, 4, 8, and 12 weeks and asked about their adherence to the activity advice. Participant in both the tele-yoga group and in the active control group were interviewed about how feasible it was to adhere to the interventions (tele-yoga or individualised activity advice) and both groups also completed an activity diary every day for 12 weeks.

The time in min each participant has taken to perform individual yoga training will be measured via statistical software incorporated in the app. Due to this software, we could analyse the number of min spent in the different sections in the app e.g., long deep breathing section and how many times the study participants used the app.

Motivation is assessed through the self-report instrument the Exercise Motivation Index [18] in both groups.

### 3.2. Acceptability of the Tele-Yoga

Acceptability was measured by questionnaire with 6 items asking about experiences of yoga, technology use including Zoom, quality of the sound, and internet and usefulness of the text and sound files in the app.

### 3.3. Safety of the Interventions (Tele-Yoga and Active Control Group)

Study participants in the tele-yoga group had the opportunity to report on adverse events (injuries, discomfort etc.) twice a week during the groups-yoga sessions with the yoga instructor. The active control group were called bi-weekly and asked about their adherence and also about adverse events.

### 3.4. Data Analysis Pilot Study

Data analysis include description of the variables (frequencies, proportions, mean values and standard deviations, medians and interquartile ranges due to the small sample size in the pilot.

## 4. Results

### 4.1. Pilot Study

Sixty-six participants were screened through chart review. Twenty-two participants were eligible and invited to participate by letter and telephone and 10 participants gave written informed consent. All participants were diagnosed with a long-term cardiac illness. All were diagnosed with heart failure on the basis of ischemic heart disease (*n* = 7), arrhythmias (*n* = 2), and hypertrophic cardiopathy (*n* = 1). They also had two or more other co-morbidities, with hypertension being the most common followed by diabetes, depression/anxiety, and cancer. For further information on the participants see Table 1.

In the pilot study the design, in terms of recruitment, enrollment, randomisation, and data collection, worked sufficiently.

### 4.2. Feasibility

#### 4.2.1. Fidelity of Tele-Yoga and Individual Activity Advice

All participants reached the goal to complete between 20 and 24 sessions during a period of 12 weeks. The recommendation for app use was 50 min per week, and a total of 600 min for the whole period. Only one participant reached this goal and above using the app in total for 960 min. Four of the participants used the app for 120 min, one used it for 180 min for the whole period.

Four of the participants in the active control group followed their individualised activity advice of doing physical activity for 60 min twice a week and a minimum of 10 min the rest of the days in the week.

#### 4.2.2. Exercise Motivation

Two participants in the yoga group increased their total exercise motivation as well as in the subscales (physical, social and psychological) and one in the active control group increased in total score and in two of the subscales (social and psychological).

### 4.3. Acceptability of the Tele-Yoga

Among the 5 participants in the tele-yoga group, one rated the overall tele-yoga experience as excellent, 3 rated very good and one as good. None had a poor or very poor experience. One participant rated the Zoom system as very easy to use, without any problems. The other four rated it as easy to use. None rated it as problematic or very problematic to use. All rated the sound on the tablet and the quality of internet during the group yoga sessions as good or very good. Four out of five found the app, both the sound files and texts, to be very useful and one as useful.

### 4.4. Safety of the Interventions and Data Collection

There were no adverse events such as injuries, acute events or mental distress caused by the different outcome measures. No participant described any negative side effect or injuries during the yoga-practice or at the interviews after finalising the tele-yoga intervention. No participant in the active control group had adverse events.

### 4.5. Primary Composite End-Point

Results on the components of the composite end-point are shown in Table 2. A change in the composite end-point included a change of 30 m in the Six Minute Walk Test (6MWT), a change in one or more dimensions of EQ-5D or being above or below a score of 8 on the HADS for anxiety and/or depression. In the tele-yoga group two participants improved in all three variables and one participant improved in two. One participant could not perform the 6MWT after 3 months. All other participants in the tele-yoga group improved 6MWT > 30 m which is considered to be clinically significant. The mean improvement in 6MWT was 57.0 ± 20.4 m. As shown in Table 2 the active control group had somewhat less favourable improvements, especially with regard to health-related quality of life and symptoms of anxiety and depression. There was one participant in the control group that could not perform the 6MWT after 3 months and one participant that choose not to participate in the 3 months follow-up. The mean improvement in 6MWT was 60.3 ± 54.0 m in the active control group.

### 4.6. Secondary End Points

#### 4.6.1. Gait Speed

Gait speed ranged between 3.90 and 7.00 s per 10-m walk at baseline in the whole group. In the tele-yoga group three participants improved their gate speed by one second and one remained unchanged. Data were missing for one participant. Gait speed in the active control group was unchanged in three participants and data were missing for two participants.

#### 4.6.2. Sit-to-Stand

The number of up-rises from sit to stand ranged between 8 to 16. One participant in the tele-yoga group deteriorated by 6 raises in the sit-to-stand test after 3 months. Two participants in the active control group improved by two raises and one deteriorated by one. The rest in both groups remained unchanged. There were missing data for 3 participants.

#### 4.6.3. Cognition

At baseline 6 of the 10 participants were mildly cognitively impaired with a score between 20 and 25 on the Montreal Cognitive Assessment (MoCA), 4 participants in the tele-yoga group and 2 in the control group. At 3 months follow-up, 1 participant in the tele-yoga group improved cognition to a level that was no longer cognitively impaired (score > 25). The rest stayed the same. Among the active controls, cognition in one participant deteriorated to become mildly impaired (score < 25), the rest stayed the same and there was one drop out.

#### 4.6.4. Health-Related Quality of Life

Two items from RAND were used. The first question was about rating the general health on a five-level numeric scale ranging from excellent to poor. None of the participants had excellent or poor health (1 or 5 on the scale). In the tele-yoga group two participants improved their general health and the other 3 stayed the same., mean difference 1.4 ± 0.57 indicated an improvement. In the active control group two stayed the same and two deteriorated, mean difference −0.5 ± 0.57 indicated a deterioration. The second item rated the health today compared to 1 year ago. After 3 months two participants in each group felt their health was much better and 3 in the tele-yoga and two in the control group that it was unchanged.

#### 4.6.5. Sleep

Sleep improved in three participants in the Tele-Yoga group and was unchanged in two. In all participants in the active control, sleep deteriorated, or was unchanged. The mean difference measured by the minimal insomnia symptom scale was 1.4 ± 2.0 (an improvement) in the tele-yoga group and −0.5 ± 1.0 (deterioration) in the active control group.

#### 4.6.6. Activity Monitoring

We collected valid data on activity monitoring in all 10 participants at baseline and 3 months. For the pilot study this data was not analysed. 

### 4.7. Process Evaluation

There were some parts of the protocol where data was collected in the pilot study, but it was not meaningful to analyse the data due to the small sample size. Interviews were analysed with regard to fidelity, but no complete inductive analyses as planned for the main RCT was done in the pilot. Health care utilisation, willingness to pay and social selectivity were also not analysed for the pilot study.

## 5. Discussion

### 5.1. Summary of Main Findings

Our pilot study showed that the tele-yoga intervention was feasible, safe, and acceptable. Furthermore, we found that the protocol for the planned RCT worked sufficiently in terms of feasibility of the screening, recruitment process, randomisation, delivery and fidelity of the tele-yoga intervention and handling of the active control group. Regarding the outcome measurements, the variables on physical function, balance, and mobility (6 min walk test , Sit to stand test , Gait speed ) had missing data at three months in two participants. Data collection on blood pressure, pulse, breathing and cognitive ability, health-related quality of life, symptoms of anxiety and depression, sleep and the Exercise Motivation as well as physical activity with an Activity Monitor had no missing data.

One person dropped out in the control group due to personal problems, not related to being randomised to the control group.

Some changes in the protocol have been made to decrease the risk of missing data in the measures of physical function during the full-scale RCT that now ongoing. The sample size calculation of 300 participants have included the estimated level of drop outs and missing data based on the pilot study.

### 5.2. Delivery of the Yoga Intervention

Standardising the yoga intervention is key, both for knowing what intervention that has been evaluated, but also for implementation. Previous studies have had large heterogeneity regarding the content of the intervention, with respect to the yoga styles used, dosage and comparison groups [21,22,23]. The strength of the current study is that we have a structured and standardised model of delivering medical yoga that makes it possible to know the exact content of the intervention delivered. Despite being structured and standardized in content, there is a flexibility in terms of how extensive and where (on a chair, in bed or on the floor) the yoga is performed. This ensures that the yoga is tailored to everyone’s health status and function.

There is a wide range of different types of yoga available which has been evaluated in previous studies [21,22,23]. There is no taxonomy of yoga although there are common elements in most yoga forms including physical postures (asanas), controlled breathing (pranayama), and meditation techniques (dhyana). To both understand effects of underlying components and facilitate implementation we standardised the tele-yoga intervention alternating between two standardised programs described in the method, which is a strength of the design.

Another aspect of the design is that when delivering tele-yoga, face-to-face contact between participants and the yoga instructor is removed. This may influence the experience, safety and outcomes of the yoga. The results from our pilot study suggest that tele-yoga is as acceptable and safe as face-to-face yoga. 

Fidelity and acceptance are important issues to consider when designing a tele-yoga study which also should be able to target individuals from all groups in the society suffering from long-term illness.

We developed a new intervention delivering medical yoga through the internet in livestreamed group sessions and an app instead of face-to-face tuition for a vulnerable population with long-term conditions. Before further evaluating the tele-yoga intervention establishing feasibility in terms of safety and acceptability was crucial. By co-designing the intervention with patients, experienced yoga therapists and an IT technician, we developed an intervention that was found to be both safe, acceptable and feasible when tested in the pilot study. The efficacy of medical yoga has been tested in different patient populations and it seems to be a promising mind–body intervention [5,24,25] However, to the best of our knowledge, there are no randomized controlled studies evaluating the effects of medical yoga in the format of tele-yoga in long-term illnesses. 

### 5.3. The Active Control Group

It is vital to consider the comparison group to evaluate the effects of tele-yoga. We decided to evaluate the tele-yoga intervention against an active control group based on ethical aspects-related guidelines for physical activity in long-term conditions, aspects of study retainment, previous experiences from earlier studies.

### 5.4. Hypothesis, Study Outcomes and Sample Size for the RCT Following the Pilot

Finally, the choice of appropriate outcomes may be challenging since this is a complex intervention [26]. This study evaluated the effect of tele-yoga with a primary outcome that combine mind and body aspects of improvement. Some patients are expected to improve their physical capacity, while others are expected to benefit from tele-yoga by reducing symptoms of anxiety and/or depression and others may benefit from tele-yoga by improving their health-related quality of life and some patients may even benefit from more than one of these aspects. A combined primary outcome including all these aspects was considered to be appropriate for this study given the fact that yoga is defined as a mind–body intervention. In addition to this advantage, by merging multiple outcomes in a common increase, the number of events decreases, thereby reducing the requirement for sample size. There are both advantages and disadvantages of using combined outcome measurements, so we will also use the separate variables in the primary outcome measure (change in 6MWT, change of HADS and change in EQ-5D) and the estimated sample size of 300 participants that we plan to recruit in the main study that have followed the pilot study will still be sufficient to analyse the variables separately. The RCT main study will recruit 300 participants until 2022 and have 6 months follow-up. The participant from the pilot study will not be included in the main RCT study.

Due to the small sample size in the pilot study, we have only described the effect in each group respectively, we did not make statistical comparisons between the groups.

We could confirm our expectations of a higher amount of missing data in tests measuring physical function, balance and walking speed (6MWT, sit-to-stand test, gate speed) since the study participants were severely ill. Fortunately, this has been taken into account when calculating the sample size and in the main RCT study we will now also inform and encourage to perform the physical test if possible.

### 5.5. Strength and Limitations

The strength of the study is that we developed a study protocol that was shown to be feasible and a co-designed tele-yoga intervention that showed promising results in feasibility, safety, and acceptability. Limitations are that there is a risk of selection bias since we only recruit study participants that has been hospitalised, not from primary care. The rationale for doing so is that persons with long term illnesses that has been hospitalized are more vulnerable and have a poorer prognosis and therefore may be in greater need for rehabilitation such as tele-yoga. There is a strength to have an active control group and controlling for attention. There might be more difficult to interpret result when not having a “pure” control group. However, based on the evidence of positive effects of physical activity in long-term illness we could not get ethical approval for a control group not given standard care e.g., exercise advice to the same extent.

## 6. Conclusions 

Yoga, in earlier studies, has been found to be a promising mind–body intervention. Our pilot study showed promising results in terms of feasibility, safety, and acceptability of tele-yoga in individuals with long-term conditions as well as feasibility of the RCT protocol.

The RCT main study that follow the pilot study will generate valuable knowledge regarding the effects of tele-yoga at home, and whether it can increase physical fitness and health-related quality of life, reduce symptoms of anxiety and depression, and improve sleep and cognition in people with long-term illness significantly compared with an active control group receiving individual training advice.

The great heterogeneity of yoga practices and the variable quality of the research done so far weakens the evidence base for recommending yoga as a therapy. Therefore, we see a great need for a well-designed and sufficiently powered study that evaluates a well-described medical yoga intervention delivered in a web-based format in a structured and standardised way.

## Figures and Tables

**Table 1:** Demographic data of the 10 participants long-term cardiac conditions in the pilot study.

	Tele-Yoga *n* = 5	Active Control *n* = 5
Age, median years (range)	50.0 (41–68)	67.0 (54–76)
Female/male gender	3/2	3/2
Married	3	5
Children	4	5
Living alone	1	0
Economic situation rated as good/problematic	4/1	5/0
Education: Secondary school/university	5/0	2/3
Computer use daily	5	5
Having a smartphone	5	3
Having a tablet	1	4
Internet use daily	5	5
Earlier experience yoga	2	2
Exercising > 3 h/week (self-reported before the study)	1	1

**Table 2:** Changes in variables included in the composite end-point. Change in depression and anxiety are merged to one score. Gray background indicates a clinically significant change defined as an improvement or deterioration of >30 m in the Six Min Walk Test (6MWT), an improvement or deterioration in one or more dimensions of EQ-5D or a score of 8 and above suggesting presence of anxiety and/or depression.

Patient	Group Randomisation	6MWT Distance Change	EQ-5D Number of the 5 Dimensions that Changed	HADS Depression	HADS Anxiety	Composite End-Point
1	Tele-yoga	+77 m	3 improved	No Depression at baseline or follow-up	Going from above to below a score of 8	+3
2	Control	+101 m	3 improved	No Depression at baseline or follow-up	Going from above to below a score of 8	+3
3	Tele-yoga	+42 m	1 improved	Going from above to below a score of 8	Going from above to below a score of 8	+3
4	Tele-yoga	+72 m	No change	No Depression at baseline or follow-up	No anxiety at baseline or follow-up	+1
5	Control	Data missing 3 months	1 improved	No Depression at baseline or follow-up	No anxiety at baseline or follow-up	+1
6	Tele-yoga	+37 m	1 improved	No Depression at baseline or follow-up	No anxiety at baseline or follow-up	+2
7	Control	+81	1 deteriorated	No Depression at baseline or follow-up	No anxiety at baseline or follow-up	0
8	Control	Drop out at 3 months				
9	Tele-yoga	Data missing 3 months	2 improved	No Depression at baseline or follow-up	No anxiety at baseline or follow-up	+1
10	Control	−1 m	No change	No Depression at baseline or follow-up	No anxiety at baseline or follow-up	−1

## Data Availability

Data is stored according to the electronic data management plan at Linköping University.
